# Temporal associations of diabetes‐related complications with health‐related quality of life decrements in Chinese patients with type 2 diabetes: A prospective study among 19 322 adults—Joint Asia Diabetes Evaluation (JADE) register (2007–2018)

**DOI:** 10.1111/1753-0407.13503

**Published:** 2023-11-20

**Authors:** Juliana N. M. Lui, Eric S. H. Lau, Aimin Yang, Hongjiang Wu, Amy Fu, Vanessa Lau, Kitman Loo, Theresa Yeung, Rebecca Yue, Ronald C. W. Ma, Alice P. S. Kong, Risa Ozaki, Andrea O. Y. Luk, Elaine Y. K. Chow, Juliana C. N. Chan

**Affiliations:** ^1^ Department of Medicine and Therapeutics, Prince of Wales Hospital The Chinese University of Hong Kong Shatin Hong Kong; ^2^ Hong Kong Institute of Diabetes and Obesity, Prince of Wales Hospital The Chinese University of Hong Kong Shatin Hong Kong; ^3^ Li Ka Shing Institute of Health Sciences The Chinese University of Hong Kong Prince of Wales Hospital Shatin Hong Kong; ^4^ Asia Diabetes Foundation Shatin Hong Kong

**Keywords:** cardiovascular renal disease, Chinese patients with type 2 diabetes, diabetes complications, HRQoL

## Abstract

**Background:**

Patients with type 2 diabetes (T2D) are at high risk of developing multiple complications, affecting their health‐related quality of life (HRQoL). Existing studies only considered impact of complication on HRQoL in the year of occurrence but not its residual impacts in subsequent years. We investigated temporal impacts of diabetes‐related complications on HRQoL in a 12‐year prospective cohort of ambulatory Chinese patients with T2D enrolled in the clinic‐based Joint Asia Diabetes Evaluation (JADE) Register.

**Methods:**

HRQoL utility measures were derived from EuroQol five‐dimensional three‐level questionnaire (EQ‐5D‐3L) questionnaires completed by 19 322 patients with T2D in Hong Kong (2007–2018). Temporal EQ‐5D utility decrements associated with subtypes of cardiovascular‐renal events were estimated using generalized linear regression model after stepwise selection of covariates with *p* < .01 as cutoff.

**Results:**

In this cohort (mean ± SD age:61.2 ± 11.5 years, 55.3% men, median [interquartile range] duration of diabetes:10.1 [3.0–15.0] years, glycated hemoglobin [HbA_1C_] 7.5 ± 1.5%), EQ‐5D utility was 0.860 ± 0.163. The largest HRQoL decrements were observed in year of occurrence of hemorrhagic stroke (−0.230), followed by ischemic stroke (−0.165), peripheral vascular disease (−0.117), lower extremity amputation (−0.093), chronic kidney disease (CKD) G5 without renal replacement therapy (RRT) (−0.079), congestive heart failure (CHF) (−0.061), and CKD G3–G4 without RRT (−0.042). Residual impacts on HRQoL persisted for 2 years after occurrence of CHF or ischemic stroke and 1 year after hemorrhagic stroke or CKD G3–G4 without RRT.

**Conclusion:**

This is the first comprehensive report on temporal associations of HRQoL decrements with subtypes of diabetes‐related complications in ambulatory Asian patients with T2D. These data will improve the accuracy of cost‐effectiveness analysis of diabetes interventions at an individual level in an Asian setting.

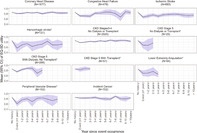

## Graphical abstract

**Highlights**

- This is the first report on health‐related quality‐of‐life (HRQoL) decrements associated with subtypes of diabetes‐related cardiovascular‐renal complications in the year of occurrence and their residual impacts in subsequent years amongst 19 322 ambulatory Asian patients with type 2 diabetes (T2D) enrolled in the clinic‐based Joint Asia Diabetes Evaluation (JADE) Register (2007–2018)
- The largest HRQoL decrements expressed as EuroQol five‐dimensional questionnaire utility scores were observed in the year of occurrence of hemorrhagic stroke (−0.230), followed by ischemic stroke (−0.165), peripheral vascular disease (−0.117), lower extremity amputation (−0.093), chronic kidney disease (CKD) G5 without renal replacement therapy (RRT) (−0.079), congestive heart failure (−0.061) and CKD G3–G4 without RRT (−0.042)
- Residual impacts on HRQoL persisted for 2 years after occurrence of CHF or ischemic stroke, and 1 year after hemorrhagic stroke or CKD G3–G4 without RRT
- HRQoL estimates considering residual impacts of diabetes complications would improve accuracy of evaluation of cost‐effectiveness of novel diabetes interventions at an individual level in an Asian setting

## 1 BACKGROUND

The global population with diabetes is predicted to increase from 537 million in 2021 to 783 million in 2045, with a quarter of them living in China.^1^ Patients with diabetes are at high risk of developing multiple co‐morbidities such as cardiovascular, renal, neurological, visual and cancer events. These events pose significant burden on healthcare systems and negatively impact patients' health‐related quality‐of‐life (HRQoL).^2^ Measurement of the latter is critical for estimating quality‐adjusted life‐years in health economic analysis. Most of the studies on HRQoL decrements related to diabetes complications were conducted in European populations.^3^, ^4^, ^5^, ^6^ Diabetes‐related complications impaired HRQoL in the year of index event and subsequent years.^7^ However, there are few prospective cohorts, such as the United Kingdom Prospective Diabetes Study, which quantified long‐term health impacts of diabetes.^8^, ^9^ Given the growing epidemic of diabetes in Asia with its cultural, economic, and healthcare systems different from the West, there is a need to investigate the long‐term temporal associations between diabetes‐related complications and HRQoL in Asian populations. Such data will provide valuable insights into the dynamic interplay between complications and HRQoL over time.

In this study, we seek to close this knowledge gap by examining temporal associations of diabetes‐related complications with HRQoL in a 12‐year prospective cohort of Chinese patients with type 2 diabetes (T2D) enrolled in the Joint Asia Diabetes Evaluation (JADE) Register in 2007–2018. These data will allow more accurate cost‐effectiveness analysis of interventions to inform long‐term policy for improving the health and HRQoL in patients with T2D.

## 2 METHODS

### 2.1 Patient sample

The Hong Kong Hospital Authority (HA) governs all public hospitals and clinics that provide 95% hospital bed‐days and 80% outpatient visits territory‐wide. All HA facilities shared a territory‐wide electronic medical record (EMR) system consisting of admission records, laboratory results, and prescriptions. The JADE Technology is a web‐based diabetes risk assessment platform established in 2007 as an extension of the Hong Kong Diabetes Register established in 1995. The latter is a quality improvement program at Prince of Wales Hospital, the teaching hospital of the Chinese University of Hong Kong (CUHK). The JADE platform includes care protocol, data entry system, and risk engines to issue personalized report with decision support. Data were recorded during nurse‐led structured assessment including medical history, eye and feet evaluation, self‐care practices, and laboratory investigations (blood and urine).^10^ In 2007, the EuroQol five‐dimensional three‐level questionnaire (EQ‐5D‐3L) HRQoL questionnaire (Traditional Chinese version) was included as an assessment item when the JADE Technology was established.

### 2.2 Inclusion and exclusion criteria

The JADE Register enrolled patients with diabetes attending two public‐hospital diabetes centers and a CUHK‐affiliated nonprofit diabetes center between 2007 and 2018. All patients underwent structured assessment using case report forms with predefined items followed by data entry into the JADE Platform (n = 22 787). Patients were referred by public and private doctors or through self‐referral.^11^ In this analysis, we excluded patients aged <18 years, non‐Chinese, and those who underwent assessment before 2007 (when EQ‐5D‐3L assessment was included) and after 2019 (when all events were censored) from the analysis. Patients with type 1 diabetes defined as continuous use of insulin within 1 year of diagnosis or presentation with acute ketosis or diabetic ketoacidosis were excluded. All patients had a unique identifier that was used to retrieve clinical outcomes based on hospitalization records in the HA EMR. All patients gave written informed consent for analysis of anonymized data and publication. The study was approved by the CUHK Clinical Research Ethics Committee (CREC number: 2007.339).

### 2.3 HRQoL assessment

EQ‐5D‐3L comprises five dimensions: mobility, self‐care, usual activities, pain/discomfort, and anxiety/depression rated by three responses: 1‐no problem, 2‐some problems, and 3‐severe problems. We included data from completed EQ‐5D‐3L surveys. EQ‐5D‐3L utility scores were reverse cross‐walked into EQ‐5D‐5L scores,^12^ then mapped to Hong Kong EQ‐5D‐5L valuation set.^13^ HRQoL utility score of 1 corresponds to full health, 0 represents a health‐state equivalent to death and negative values imply health‐states worse than death.

### 2.4 Temporality of diabetes complications

Diabetes complications of interest included coronary heart disease (CHD), congestive heart failure (CHF), ischemic stroke, hemorrhagic stroke, peripheral vascular disease (PVD), lower extremity amputation (LEA), end‐stage kidney disease (ESKD), and any type of cancer. Events were defined by International Classification of Disease, Ninth Revision codes of all admissions and procedures^14^ (Supplementary Table S1).

To estimate temporal impacts of events on HRQoL, ordered time categories were generated with reference to time period between date of complication event closest to the date of structured assessment and EQ‐5D‐3L survey. Time categories were assigned as “no event,” event experienced “within 1 year,” “within 2 to 3 years,” “within 3 to 4 years,” “within 4 to 5 years,” and “more 5 five years” prior to the measurement of HRQoL. For each complication, time categories with ≤5 events were combined with previous time category level. Consecutive time categories (adjusted for patients' personal and clinical characteristics) without significant differences were combined (F‐test at 1% significance level).

### 2.5 Definitions and covariates

T2D was defined as lack of history of ketoacidosis or nonrequirement of continuous insulin treatment within 1 year of diagnosis. Variables including demographics, lifestyle, types of care (public/ private), clinical and biochemical characteristics, complications, medical treatments, and histories were adjusted in our model. All data were collected in structured case report forms with codes and definitions as listed in Supplementary Table S2.

### 2.6 Statistical analysis

#### 2.6.1 Specification of regression models

Data are expressed as mean (SD) or median (interquartile range). HRQoL regression was modeled using generalized linear model with Gaussian family distribution and linear link function, adjusting for patients' demographic and clinical characteristics, temporal diabetes complication events and Elixhauser Comorbidity Index (ECI).^15^

Stepwise selection of covariates was carried out to evaluate associations of HRQoL with temporality of diabetes complications and other risk factors. Covariates including age, sex, duration of diabetes, year of assessment, smoking habit, and drug use were kept stable during covariate selection. Covariates with significance at 1% level were included in the final model. Missing data were imputed for 10 iterations. All analyses were conducted using R 3.4.2. A P‐value<0.01 was considered significant.

## 3 RESULTS

Of 22 787 Chinese patients with T2D enrolled in the JADE Register between 2007 and 2018, 19 322 (84.8%) completed all items in the EQ‐5D‐3L questionnaire. Table 1 summarizes the clinical profile of these patients, of whom 55.3% were men. The mean age of the cohort was 61.2 years (SD: 11.5); mean age of diagnosis, 51.1 years (SD:11.8), and mean diabetes duration, 10.1 years (interquartile range: 3.0–15.0). More than half attended middle school or above (58.8%) and had regular physical activity (57.4%). Nearly one third were occasional or regular alcohol drinkers (28.5%) and 1 in 10 patients were current smokers (11.4%). Over half were overweight or obese (body mass index [BMI]≥25 kg/m^2^; 55.3%), had a diastolic blood pressure (BP) ≥75 mm Hg (55.5%), fasting plasma glucose (FPG) ≥7 mmol/L (54.4%), or HbA_1C_ ≥ 7.0% (57.7%. One in five patients had reduced kidney function with estimated glomerular filtration rate (eGFR) ≤60 mL/min/1.73m^2^ (20.8%). The majority of patients were on glucose‐lowering drugs (GLDs, 85.7%), 26.2% on insulin, 52.9% on statins, 35.8% on angiotensin‐converting enzyme (ACE) inhibitors, 15.3% on angiotensin receptor blocker (ARB), and 19.9% on BP lowering drugs (excluding ACE inhibitors/ARB). Most patients (87.6%) visited public hospital clinics for baseline assessment. One in four patients (21.3%) self‐reported having experienced hypoglycemia in the last 3 months.

**TABLE 1:** Clinical profiles of 19 322 patients with T2D during structured assessment enrolled in the JADE Register in 2007–2018.

Variable	Mean or *n* (SD or %)
Personal characteristics	
Men (*n* [%])	10 678 (55.3)
Age at assessment (years) (*n* [%])	61.2 (11.5)
<40 years old	796 (4.1)
40–59 years old	7870 (40.7)
≥60 years old	10 656 (55.1)
Age at diabetes diagnosis (years) (*n* [%])	51.1 (11.8)
<40 years old	3055 (15.8)
40–59 years old	11 770 (60.9)
≥60 years old	4383 (22.7)
Missing	114 (0.6)
Duration of diabetes (years) (*n* [%])	10.1 (8.4)
<5 years	6180 (32.0)
5–9 years	4388 (22.7)
10–14 years	3574 (18.5)
≥15 years	5064 (26.2)
Missing	116 (0.6)
Type of care model	
Public	16 918 (87.6)
Private	2053 (10.6)
Missing	351 (1.8)
Year of structured assessment with EQ‐5D‐3 L data (year) [mean (SD)]	2012.76 (2.32)
Education (*n* [%])	
Primary, illiterate or others	7897 (40.9)
Middle or high school	9052 (46.8)
College or above	2319 (12.0)
Missing	54 (0.3)
Lifestyle factors	
Alcohol use (*n* [%])	
Never	11 446 (59.2)
Occasional	4889 (25.3)
Regular	616 (3.2)
Ex‐drinker	2335 (12.1)
Missing	36 (0.2)
Smoking (*n* [%])	
Never	13 124 (67.9)
Current smoker	2196 (11.4)
Ex‐smoker	3985 (20.6)
Missing	17 (0.1)
Physical activity (per week) (*n* [%])	
No regular activity	7442 (38.5)
<3 times	2654 (13.7)
3–4 times	1475 (7.6)
5 times	812 (4.2)
>5 times	6173 (31.9)
Missing	766 (4.0)
Frequency of hypoglycemia (*n* [%])	
At least daily	30 (0.2)
At least once monthly	1288 (6.7)
At least once weekly	460 (2.4)
Less than once monthly	2322 (12.0)
None	15 156 (78.4)
Missing	66 (0.3)
Clinical characteristics	
Body mass index (kg/m^2^) (*n* [%])	26.1 (4.4)
<25	8561 (44.3)
25–30	7593 (39.3)
≥30 or more	3095 (16.0)
Missing	73 (0.4)
Diastolic blood pressure (mm Hg) (*n* [%])	76.5 (11.0)
<75	8574 (44.4)
≥75 < 85	6557 (33.9)
≥85	4182 (21.6)
Missing	9 (0.0)
Visual acuity (*n* [%])	
No significant visual impairment	18 495 (95.1)
Severe visual impairment (counting fingers, hand movement, light perception)	821 (4.2)
Blind (no light perception)	125 (0.6)
Complications and comorbidities	
Sensory neuropathy (*n* [%])	
True	1211 (6.3)
Diabetic retinopathy	
None	14 249 (73.3)
Nonproliferative/ preproliferative retinopathy	4668 (24.0)
Proliferative retinopathy, maculopathy, or advanced eye disease	524 (2.7)
History of retinal, cataract surgery, or laser treatment	
True	3513 (18.1)
Renal disease	
CKD G3–G4 (No dialysis or transplant) (*n* [%])	
< 1 year	1255 (6.5)
≥1 year	1665 (8.6)
CKD G5 (No dialysis or transplant) (*n* [%])	
Any year	29 (0.2)
Cardiovascular disease	
Congestive heart failure (*n* [%])	
<1 year	164 (0.8)
≥1 < 2 years	104 (0.5)
≥2 < 3 years	71 (0.4)
≥3 years	139 (0.7)
Ischemic stroke (*n* [%])	
<1 year	135 (0.7)
≥1 < 2 years	83 (0.4)
≥2 years	475 (2.5)
Hemorrhagic stroke (*n* [%])	
<1 year	25 (0.1)
≥1 year	106 (0.5)
Peripheral vascular disease (*n* [%])	
<1 year	44 (0.2)
≥1 year	108 (0.6)
Lower extremity amputation (*n* [%])	
Any year	94 (0.5)
Elixhauser comorbidity score (van Walraven 2009) (*n* [%])	
< 0	550 (2.8)
0	13 202 (67.9)
>0 < 5	1482 (7.6)
≥5 < 15	3376 (17.4)
≥15 < 25	768 (4.0)
≥25	63 (0.3)
Biochemistry	
Fasting plasma glucose (mmol/L)) (*n* [%])	7.7 (2.6)
<5.7	3253 (16.8)
≥5.7 < 7.0	5480 (28.4)
≥7.0	10 502 (54.4)
Missing	87 (0.5)
Hemoglobin A1C (%) (*n* [%])	7.5 (1.5)
<5.7	617 (3.2)
≥5.7 < 7.0	7516 (38.9)
≥7.0	11 153 (57.7)
Missing	36 (0.2)
HDL‐cholesterol (mmol/L) (*n* [%])	1.3 (0.4)
<1.3	9483 (49.1)
≥1.3 < 1.55	5502 (28.5)
≥1.55	4228 (21.9)
Missing	109 (0.6)
LDL‐cholesterol (mmol/L) (*n* [%])	2.4 (0.8)
<2.6	11 483 (59.4)
≥2.6 < 3.35	5100 (26.4)
≥3.35	2334 (12.1)
Missing	405 (2.1)
Estimated glomerular filtration rate (mL/min/1.73m^2^) (*n* [%])	78.9 (24.6)
<30	956 (4.9)
≥30 < 60	3064 (15.9)
≥60 < 90	7633 (39.5)
≥90	7648 (39.6)
Missing	21 (0.1)
Alanine transaminase levels (IU/L) (*n* [%])	28.3 (22.5)
<10	473 (2.4)
≥10 < 40	15 638 (80.9)
≥40	3109 (16.1)
Missing	102 (0.5)
Plasma albumin (g/L) (*n* [%])	43.5 (3.43)
<35	317 (1.6)
≥35 < 50	18 586 (96.2)
≥50	334 (1.7)
Missing	85 (0.4)
Hemoglobin (g/dL) (*n* [%])	13.4 (1.6)
<10	519 (2.7)
≥10	18 650 (96.5)
Missing	153 (0.8)
Treatments (Ref: none)	
Glucose lowering drugs (*n* [%])	
True	16 563 (85.7)
Insulin (*n* [%])	
True	5054 (26.2)
Statins (*n* [%])	
True	10 233 (52.9)
Missing	258 (1.3)
Angiotensin‐converting enzyme (ACE) inhibitor (*n* [%])	
True	6911 (35.8)
Missing	190 (1.0)
Angiotensin receptor blocker (ARB) (*n* [%])	
True	2961 (15.3)
Missing	190 (1.0)
Blood pressure lowering drugs (excluding RAS inhibitors—ACE/ARB) (*n* [%])	
True	3853 (19.9)
Missing	191 (1.0)

Abbreviations: CKD, chronic kidney disease; EQ‐5D‐3L, EuroQol five‐dimensional three‐level questionnaire; HDL, high‐density lipoprotein; LDL, low‐density lipoprotein; RAS, renin‐angiotensin system; T2D, type 2 diabetes.

Among the EQ‐5D domains, 29.9% of patients reported some or severe problems in pain/discomfort, 18.9% for anxiety/depression, 9.5% for mobility, 6.7% for usual activity, and 3.2% for self‐care (Table 2). The mean EQ‐5D utility score was 0.860 (SD: 0.163) and 11 450 (59.3%) reported full health. The mean EQ‐5D utility of patients who did not experience any complications was 0.869 (SD: 0.149) and decreased to 0.792 (SD: 0.227) in those who experienced one complication and 0.724 (SD: 0.290) in those who experienced two or more complications.

**TABLE 2:** EuroQoL five‐dimensional questionnaire responses from 19 322 participants.

	Mobility (*n* [%])	Self‐care (*n* [%])	Usual activities (*n* [%])	Pain/discomfort (*n* [%])	Anxiety/depression (*n* [%])
1 No problem	17 485 (90.5)	18 713 (96.8)	18 038 (93.4)	13 547 (70.1)	15 658 (81.0)
2 Some problems	1786 (9.2)	495 (2.6)	1118 (5.8)	5366 (27.8)	3485 (18.0)
3 Severe problems	51 (0.3)	114 (0.6)	166 (0.9)	409 (2.1)	179 (0.9)

**FIGURE 1:**
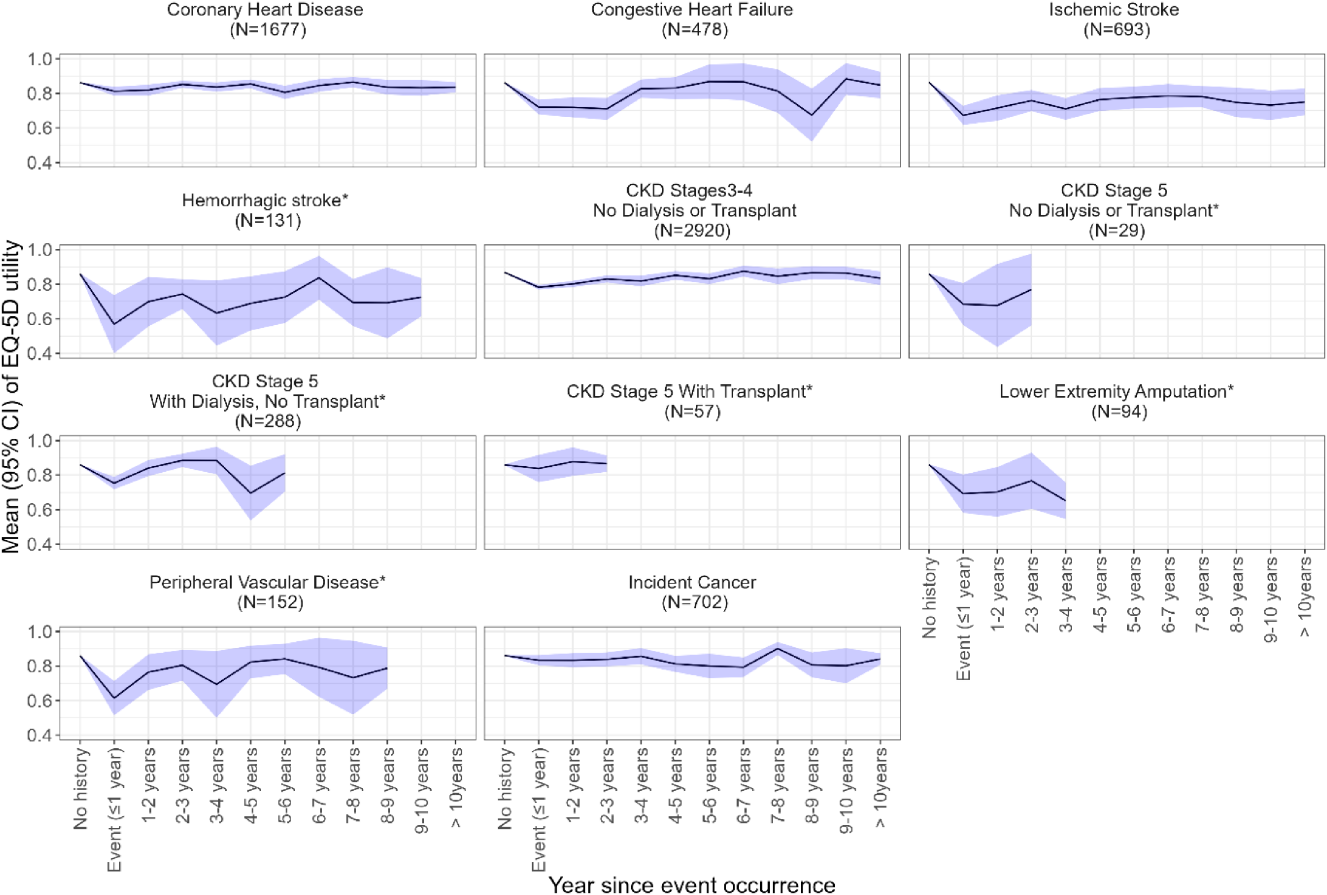
EuroQoL five‐dimensional (EQ‐5D) utility by adverse event and time since event occurrence. *Too few participants experienced an event in further years prior to EQ‐5D response, thus EQ‐5D utility were aggregated for participants who had experienced an event 9 years ago or more for hemorrhagic stroke, 2 years ago or more for CKD G5 (no dialysis or transplant), 5 years ago or more for CKD G5 (with dialysis, no transplant), 2 years ago or more for CKD G5 (with transplant and dialysis), and 3 years ago or more for lower extremity amputation. CI, confidence interval; CKD, chronic kidney disease.

Unadjusted mean HRQoL utility trends by time of occurrence for each complication are summarized in Figure 1. Large decrements in HRQoL utility were observed in year of occurrence of hemorrhagic stroke, ischemic stroke, PVD, LEA, and chronic kidney disease (CKD) G5 without renal replacement therapy (RRT). Although EQ‐5D utilities gradually improved over time, it rarely recovered to the utility levels before the occurrence of event.

### 3.1 EQ‐5D decrements associated with diabetes‐related complications

**FIGURE 2:**
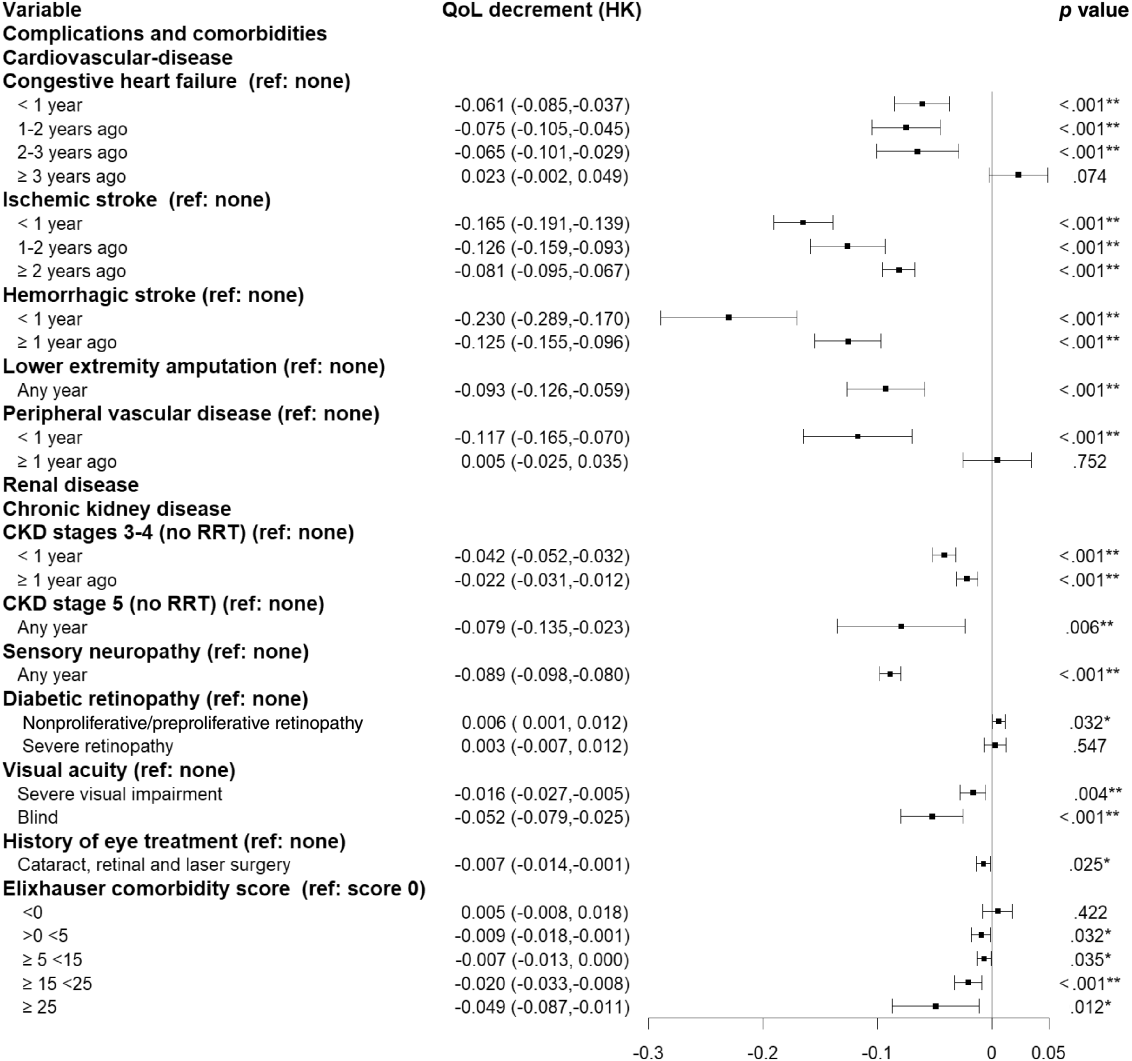
EuroQoL five‐dimensional (EQ‐5D) utility decrements in people with diabetes associated with complications. CKD, chronic kidney disease; HK, Hong Kong; QoL, quality of life; RRT, renal replacement therapy.

Figure 2 shows the temporal associations of EQ‐5D utility decrements with diabetes‐related complications. For cardiovascular events, the largest EQ‐5D utility decrements were observed in year of occurrence of a hemorrhagic stroke (−0.230 [95% confidence interval (CI): −0.289 to −0.170]), followed by ischemic stroke (−0.165, [95% CI: −0.191 to −0.139]), PVD (−0.117 [95% CI: −0.165 to −0.070]), LEA in any year after occurrence (−0.093 [95% CI: −0.126 to −0.059]), and CHF (−0.061 [95% CI: −0.085 to −0.037]).

For kidney events, occurrence of CKD G5 without RRT in any year after event occurrence was associated with greater HRQoL decrement (−0.079, [95% CI: −0.135 to −0.023]) compared to occurrence of CKD G3–G4 without RRT (−0.042 [95% CI: −0.052 to −0.032]). We examined the changes in eGFR and urinary albumin creatinine ratio (ACR) values 3 years before and 3 years after their first event of hospitalization with chronic kidney disease as the primary diagnosis. Patients showed significant improvements in mean eGFR and ACR after transplantation as compared to progressive decline in eGFR and increase in ACR in patients at G3–G4 or G5 without receiving renal replacement therapy (Supplementary Figure S1).

Detection of sensory neuropathy during assessment was associated with worse HRQoL decrement than those with visual problems. For the latter, blindness was associated with the worst HRQoL decrement, followed by severe visual impairment and history of eye treatment. Because EQ‐5D utility was not impaired in patients with diabetes who had CHD, cancer, and CKD G5 with RRT, these complications were not included in our final model.

In most patients, cardiovascular event had significant residual impacts on HRQoL that seldom returned to preevent health‐state. Ischemic stroke or CHF had significant detrimental impacts on HRQoL that persisted for 2 years and remained static. HRQoL decrements were also observed 1 year after occurrence of CKD G3–G4 without RRT. Patients with increasing number of comorbidities (higher ECI scores) had worse HRQoL (Figure 2).

### 3.2 EQ‐5D utility decrements associated with demographics, risk factors, and behaviors

Independent of occurrence of these complications, female sex, age of diabetes diagnosis ≥60 years, ≥15 years of diabetes, BMI ≥30 kg/m^2^, and being ex‐alcohol drinkers were associated with lower HRQoL (Supplementary Table S3). Frequent self‐reported hypoglycemia episodes were associated with greater HRQoL decrements whereas frequent physical activity levels were associated with better HRQoL. Patients with lower education level (primary, illiterate, or others: ≤6 years) had worse HRQoL than those with higher level (middle or high school: >6 and ≤13 years).

Users of GLDs and ACE inhibitors had better HRQoL than nonusers. Insulin use was not associated with significant HRQoL decrement (Supplementary Table S3). For risk factors, low‐density lipoprotein (LDL)‐cholesterol ≥2.6 mmol, HbA_1C_ ≥5.7%, FPG ≥5.7 mmol/L, and eGFR 30–60 mL/min/1.73m^2^ were associated with higher HRQoL and high‐density lipoprotein (HDL)‐cholesterol <1.3 mmol/L and diastolic BP ≥75 mm Hg were associated with reduced HRQoL utilities. Patients with serum albumin<35 g/L, alanine transaminase<10 IU/L, and hemoglobin <10 g/dL were associated with reduced HRQoL.

## 4 DISCUSSION

This is the first report on the temporal associations of a comprehensive array of subtypes of diabetes‐related cardiovascular‐renal complications on HRQoL using the prospective data from the JADE Register. During a 12‐year period, 19 322 Chinese patients with T2D from Hong Kong enrolled in the register and completed the EQ‐5D survey. Cardiovascular‐renal complications had detrimental and long‐lasting impacts on HRQoL. The largest HRQoL decrement expressed as EQ‐5D utility score was observed in year of occurrence of a hemorrhagic stroke (−0.230), followed by ischemic stroke (−0.165), PVD (−0.117), LEA (−0.093), sensory neuropathy (−0.089), CKD G5 without RRT (−0.079), CHF (−0.061), and CKD G3–G4 without RRT (−0.042). Residual impacts on HRQoL persisted after index event of CHF, ischemic stroke, hemorrhagic stroke, or CKD G3–G4 without RRT. However, CHD, cancer, and CKD G5 with RRT were not associated with impaired HRQoL.

In alignment with other studies, 20%–30% of JADE participants reported some problems with pain/discomfort and anxiety/depression.^16^ The mean EQ5D utility of 0.860 from our cohort was comparable to that reported in Asian patients with diabetes (0.84–0.91).^16^, ^17^, ^18^, ^19^, ^20^ In general, these scores were higher in Asian than their European counterparts (0.72–0.80) possibly due to differences in values, culture, and traditions.^16^, ^21^, ^22^ Although HRQoL decrements related to diabetes complications had been reported in Asians, its temporal relationship with time of occurrence of event has not been previously estimated.^20^, ^23^, ^24^, ^25^ Our study is the first to evaluate residual HRQoL decrements related to diabetes complications in subsequent years after occurrence of the event. These prospective data will allow more precise estimation of health benefits of new diabetes interventions and their long‐term cost‐effectiveness.

Previous studies in Asia that explored HRQoL decrements with diabetes complications had considerably less granularity than our report.^18^, ^24^, ^26^, ^27^, ^28^ For example, this is the first Asian study to report HRQoL decrements for ischemic and hemorrhagic stroke subtypes. In a meta‐analysis, sodium‐glucose‐transporter 2 inhibitors reduced hemorrhagic stroke but not ischemic stroke or all‐stroke.^29^ By contrast, glucagon‐like‐peptide receptor agonist reduced ischemic stroke but not hemorrhagic stroke.^30^ Given subtle differences in etiologies and risk factors for stroke subtypes as well as drug mechanisms, differences in drug effects are plausible. These HRQoL estimates enable us to identify patient subgroups who will benefit most from a drug using cost‐effectiveness models simulated using individual patient data.

Although the overall HRQoL of Asian patients with T2D was higher than that of their European counterparts,^31^, ^32^, ^33^ Asian patients were more prone to develop stroke than CHD.^34^ In this study, ischemic (−0.165) and hemorrhagic stroke (−0.230) decrements were higher than that reported in studies with mainly Europeans (ASCEND [A Study of Cardiovascular Events iN Diabetes] trial ischemic stroke: −0.062,^7^ LEADER [Liraglutide Effect and Action in Diabetes: Evaluation of Cardiovascular Outcome Results] trial stroke: −0.046,^32^ ADVANCE [Action in Diabetes and Vascular Disease‐PreterAx and DiamicroN Controlled Evaluation] trial stroke: −0.099,^5^ and MEPS [Medical Expenditure Panel Survey] database stroke: −0.060).^35^ Our findings corroborate results from the Acarbose Cardiovascular Evaluation trial conducted in Chinese patients with impaired glucose tolerance (IGT) (overall stroke: −0.107)^36^ and a hospital‐based study in Chinese patients with T2D (overall stroke: −0.101).^18^ In this trial, which enrolled patients with prior cardiovascular disease and IGT, HRQoL decrements associated with stroke were higher than those in European patients with T2D. To this end, high prevalence of stroke in Asians might be due to high salt intake and high prevalence of hypertension, further aggravated by increasing obesity and diabetes, related to rapid socioeconomical and lifestyle transition.^37^ These regional, ecological, and cultural differences call for more effective stroke prevention strategies to reduce the immediate and residual impacts of stroke on HRQoL in Asians with T2D.^38^, ^39^

In our study, patients who experienced CHF had persistent HRQoL decrements in subsequent years. This contrasts the improvements in QoL decrements in subsequent years reported in the ACCORD trial (<1 year: −0.089, >1 year ago: −0.041).^4^ HRQoL decrements related to PVD (−0.117) were comparable to Japanese (−0.070)^28^ and European (−0.061)^40^ patients with diabetes. In our cohort, LEA HRQoL decrements (−0.093) were smaller than in other populations (Japan: −0.178,^28^ United Kingdom: −0.272,^40^ ASCEND trial: −0.206^7^), which may be due to the majority of our patients having minor toe amputations only.

Our reported HRQoL decrements for ESKD (G5 without RRT) (−0.079) were similar to other reports (ADVANCE trial: −0.049,^5^ Japanese cohort: −0.050^28^). In our analysis, patients undergoing RRT exhibited significant improvements in albuminuria and eGFR, in contrast to those who did not receive RRT, who experienced a progressive decline in kidney function. Compared to patients at G3–G4 without RRT, patients at CKD G5 with RRT had no significant HRQoL decrements. Other researchers had reported considerable improvement in HRQoL in patients with ESKD after RRT, especially with transplantation, the latter being considered a cost‐effective treatment in many countries.^41^, ^42^

Decrements in HRQoL associated with sensory neuropathy (−0.089) in this cohort were comparable to similar reports (Europe: −0.084,^40^ China: −0.057,^25^ Japan: −0.066^28^). For proliferative retinopathy and visual acuity, the HRQoL decrements were similar to those in Japanese (blindness in one eye: −0.032, blindness in both eyes: −0.108)^28^ and Chinese (sight‐threatening retinopathy: −0.043)^24^, ^25^ cohorts. These data highlighted that, although these microvascular complications might not be immediately life threatening, their negative impacts on HRQoL should not be underestimated and call for better prevention efforts.^43^ In agreement with other studies, multimorbidity indicated by increased ECI scores was associated with worse HRQoL (score ≥25: −0.049). Although other local researcher had reported greater decrements with increasing number of diabetes comorbidities (three: −0.060, ≥four: −0.173), they had not adjusted for the impacts of diabetes‐related complications.^20^

Concurring with other reports,^7^, ^24^, ^26^, ^44^ we did not observe significant HRQoL decrements with CHD, which might be attributed to advancements in cardiology‐related technology. The lack of significant HRQoL decrements with incident cancers and CKD G5 with RRT might be due to referral or survival bias. Patients in the JADE Register were assessed in an ambulatory care setting, which might have limited the representation of patients with severe diseases. Because patients on RRT or those with cancer were followed up frequently at dialysis and cancer centers respectively, they were less likely to be referred to the diabetes center for assessment and education with possible referral bias.

The associations of female gender,^7^, ^20^, ^23^, ^28^ old age,^5^, ^7^, ^23^ low education level,^27^ obesity,^7^, ^23^ and alcohol use^25^ with low HRQoL were also observed in our cohort whereas patients with exercise ≥ three times weekly had higher HRQoL. In the Look AHEAD (Action for Health in Diabetes) trial, 175 min exercise weekly with ≥7% weight loss improved not only HRQoL but also control of blood glucose, BP, and blood lipids accompanied by reduced incidence of CKD, retinopathy, and depression.^45^ Physical exercise is known to increase secretion of neurotransmitters such as endocannabinoids^46^ and brain‐derived neurotrophic factor,^47^ which might reduce depression and anxiety.

In our analysis, LDL‐cholesterol ≥2.6 mmol, HbA_1C_ ≥5.7%, or eGFR 30–60 mL/min/1.73 m^2^ were associated with better HRQoL. We speculate that high fat and sugary diets might upregulate the reward pathway and alter neurochemical transmitters (dopamine, serotonin) with increased sense of well‐being and pleasure.^48^ These associations of LDL‐cholesterol and HbA_1C_ levels with HRQoL were independent of complications. Although patients on high fat and sugar diets might feel fit, over time, suboptimal risk factor control will be translated to complications with HRQoL reductions. Patients with HDL‐cholesterol <1.3 mmol/L and diastolic BP ≥75 mm Hg (markers of obesity) had suboptimal HRQoL. Parameters of liver dysfunction (low albumin), often associated with chronic poor‐health,^49^ was also associated with decreased HRQoL.

Despite potential side effects, use of oral GLDs and ACE inhibitors was associated with better HRQoL than nonuse. In line with previous reports,^50^ HRQoL decrements were associated with increasing frequency of self‐reported hypoglycemia episodes. Similar to the Dutch SPIRIT study,^51^ there was no difference in HRQoL between insulin and noninsulin users. In our study, patients treated with ARB also had better HRQoL than nonusers. Apart from proven organ‐protective effects, ARB had neutral side effects compared to other BP‐lowering drugs, which could limit their effectiveness due to poor adherence.^52^

Our study is not without limitations. Patients with lower baseline HRQoL might experience further decrements with occurrence of events although we did not have previous HRQoL measures for comparison. Over 80% of patients were attending hospital‐based clinics and our results might not be representative of patients receiving primary care. Survival bias was possible as participants who died or those who were too ill would not undergo these assessments. That said, our results were largely in agreement with similar studies, with temporal associations as a novel feature.

Our study has multiple strengths. This is the first large‐scale, prospective study in Asian patients with T2D spanning for 12 years. A real‐world cohort has considerably less volunteer bias compared to participants of randomized controlled trials (RCTs). In this real‐world register, we have accrued large number of events that could not have been captured in expensive RCTs with short follow‐up duration. This analysis was based on patients enrolled from a single region. This had reduced regional variations in HRQoL rating as reported in multinational RCTs^5^, ^44^ or meta‐analysis.^53^

## 5 CONCLUSIONS

In conclusion, our study provides temporal associations of HRQoL decrement with a comprehensive array of subtypes of cardiovascular‐renal events in Chinese patients with T2D. By incorporating these temporal HRQoL estimates indicating the residual impacts of an index event in a simulation model, we can provide accurate estimation of the long‐term cost‐effectiveness of innovative interventions on an individual patient level in an Asian setting.

## AUTHOR CONTRIBUTIONS

Juliana Lui, Juliana Chan, and Elaine Chow contributed to the study design. Juliana Lui conducted the analysis and drafted the manuscript. Juliana Chanis the principal investigator of the JADE Register and provided important clinical insights and results interpretation throughout the study. Eric Lau is the data manager of JADE and contributed to data curation and data cleaning. All authors contributed to data collection, interpretation of results, and revision of the manuscript and approved the final version for submission.

## CONFLICT OF INTEREST STATEMENT

Juliana Chan reported receiving grants and/or honoraria for consultancy or giving lectures from Applied Therapeutics, AstraZeneca, Bayer, Boehringer Ingelheim, Celltrion, Eli Lilly, Hua Medicine, Powder Pharmaceuticals, Merck, MSD, Pfizer, Sanofi, Servier, Viatris, and Zuelig Pharma. She is the chief executive officer (pro bono) of the Asia Diabetes Foundation that developed the web‐based JADE platform for implementation of data‐driven diabetes care. She holds patents for using biomarkers to predict diabetes and its complications. She is the cofounder of GemVCare, a biotech company, with partial support from the Hong Kong Government, which uses biogenetic markers and information technology to implement precision diabetes care and prevention through partnerships. Juliana Chan is a member of the editorial board of the *Journal of Diabetes* and a coauthor of this article. To minimize bias, she was excluded from all editorial decision‐making related to the acceptance of this article for publication. Alice Kong has received honorarium for consultancy or giving lectures from Abbott, Astra Zeneca, Bayer, Boehringer Ingelheim, Eli‐Lilly, Kyowa Kirin, Merck Serono, Nestle, Novo‐Nordisk, Pfizer, and Sanofi. Andrea Luk has received research grants and/or honorarium and/or served as a member of advisory panel for Amgen, AstraZeneca, Bayer, Boehringer Ingelheim, Lee's Pharmaceutical, MSD, Novo Nordisk, Roche, Sanofi, Sugardown Ltd, and Takeda, outside the submitted work. Ronald Ma has received research grants and/or honoraria for consultancy or giving lectures from AstraZeneca, Boehringer Ingelheim, Bayer, Kyowa Kirin, Merck, Novo Nordisk, Pfizer, Roche Diagnostics, and Tricida Inc. The proceeds have been donated to the Chinese University of Hong Kong, American Diabetes Association, and other charitable organizations to support diabetes research and education. The other authors declare no conflict of interest.

## CONSENT FOR PUBLICATION

All authors have given consent for publication.

## Supporting information


**Table S1.** International Classification of Diseases, Ninth Revision (ICD‐9) admissions and procedure codes for categorization of diabetes complication.
**Table S2.** Definitions of covariates included for selection.
**Table S3.** EuroQol 5‐dimensional questionnaire (EQ‐5D) utility in patient with diabetes.
**Figure S1.** Annual mean estimated glomerular filtration rate (eGFR) and urinary albumin creatinine ratio (UACR) of patients with diabetes who experience different stages of chronic kidney disease.

## References

[1] International Diabetes Federation . IDF Diabetes Atlas. 10th ed. International Diabetes Federation; 2021.

[2] Rubin RR , Peyrot M . Quality of life and diabetes. Diabetes Metab Res Rev. 1999;15(3):205‐218. 10441043 10.1002/(sici)1520-7560(199905/06)15:3<205::aid-dmrr29>3.0.co;2-o

[3] Alva M , Gray A , Mihaylova B , Clarke P . The effect of diabetes complications on health‐related quality of life: the importance of longitudinal data to address patient heterogeneity. Health Econ. 2014;23(4):487‐500. 23847044 10.1002/hec.2930

[4] Shao H , Yang S , Fonseca V , Stoecker C , Shi L . Estimating quality of life decrements due to diabetes complications in the United States: the health utility index (HUI) diabetes complication equation. Pharmacoeconomics. 2019;37:921‐929. 30778865 10.1007/s40273-019-00775-8 PMC7220804

[5] Hayes A , Arima H , Woodward M , et al. Changes in quality of life associated with complications of diabetes: results from the ADVANCE study. Value Health. 2016;19(1):36‐41. 26797234 10.1016/j.jval.2015.10.010

[6] Beaudet A , Clegg J , Thuresson P‐O , Lloyd A , McEwan P . Review of utility values for economic modeling in type 2 diabetes. Value Health. 2014;17(4):462‐470. 24969008 10.1016/j.jval.2014.03.003

[7] Keng MJ , Leal J , Bowman L , Armitage J , Mihaylova B , Group ASC . Decrements in health‐related quality of life associated with adverse events in people with diabetes. Diabetes Obes Metab. 2022;24(3):530‐538. 34866309 10.1111/dom.14610 PMC9361007

[8] Hayes AJ , Leal J , Gray A , Holman R , Clarke P . UKPDS outcomes model 2: a new version of a model to simulate lifetime health outcomes of patients with type 2 diabetes mellitus using data from the 30 year United Kingdom prospective diabetes study: UKPDS 82. Diabetologia. 2013;56:1925‐1933. 23793713 10.1007/s00125-013-2940-y

[9] Holman RR , Paul SK , Bethel MA , Matthews DR , Neil HAW . 10‐year follow‐up of intensive glucose control in type 2 diabetes. N Engl J Med. 2008;359(15):1577‐1589. 18784090 10.1056/NEJMoa0806470

[10] Ko GT , So WY , Tong PC , et al. From design to implementation – the joint Asia diabetes evaluation (JADE) program: a descriptive report of an electronic web‐based diabetes management program. BMC Med Inform Decis Mak. 2010;10:26. doi:10.1186/1472-6947-10-26 20465815 PMC2876072

[11] Lim LL , Lau ES , Ozaki R , et al. Association of technologically assisted integrated care with clinical outcomes in type 2 diabetes in Hong Kong using the prospective JADE program: a retrospective cohort analysis. PLoS Med. 2020;17(10):e1003367. 33007052 10.1371/journal.pmed.1003367 PMC7531841

[12] van Hout B , Janssen MF , Feng Y‐S , et al. Interim scoring for the EQ‐5D‐5L: mapping the EQ‐5D‐5L to EQ‐5D‐3L value sets. Value Health. 2012;15(5):708‐715. doi:10.1016/j.jval.2012.02.008 22867780

[13] Wong ELY , Ramos‐Goñi JM , Cheung AWL , Wong AYK , Rivero‐Arias O . Assessing the use of a feedback module to model EQ‐5D‐5L health states values in Hong Kong. Patient. 2018;11(2):235‐247. doi:10.1007/s40271-017-0278-0 29019161 PMC5845074

[14] Wu H , Lau ES , Yang A , et al. Data resource profile: the Hong Kong diabetes surveillance database (HKDSD). Int J Epidemiol. 2022;51(2):e9‐e17. 34904159 10.1093/ije/dyab252

[15] Elixhauser A , Steiner C , Harris DR , Coffey RM . Comorbidity measures for use with administrative data. Med Care. 1998;36(1):8‐27. 9431328 10.1097/00005650-199801000-00004

[16] Afshari S , Ameri H , Baharinya S , Arab‐Zozani M , Mojahedian MM . Assessment of the properties of the EQ‐5D‐5L in patients with type 2 diabetes mellitus: a systematic review and meta‐analysis. Expert Rev Pharmacoecon Outcomes Res. 2022;22(2):351‐364. 35012416 10.1080/14737167.2022.2011216

[17] Guo X , Wong PNF , Koh YLE , Tan NC . Factors associated with diabetes‐related distress among Asian patients with poorly controlled type‐2 diabetes mellitus: a cross‐sectional study in primary care. BMC Prim Care. 2023;24(1):1‐11. 36849921 10.1186/s12875-023-02012-w PMC9969642

[18] Zhang Y , Wu J , Chen Y , Shi L . EQ‐5D‐3L decrements by diabetes complications and comorbidities in China. Diabetes Ther. 2020;11:939‐950. 32152932 10.1007/s13300-020-00788-z PMC7136375

[19] Jeong M . Predictors of health‐related quality of life in Korean adults with diabetes mellitus. Int J Environ Res Public Health. 2020;17(23):9058. 33291678 10.3390/ijerph17239058 PMC7730541

[20] Wong ELY , Xu RH , Cheung AWL . Measurement of health‐related quality of life in patients with diabetes mellitus using EQ‐5D‐5L in Hong Kong, China. Qual Life Res. 2020;29:1913‐1921. 32140920 10.1007/s11136-020-02462-0 PMC7295714

[21] Zhang P , Brown MB , Bilik D , Ackermann RT , Li R , Herman WH . Health utility scores for people with type 2 diabetes in U.S. managed care health plans: results from translating research into action for diabetes (TRIAD). Diabetes Care. 2012;35(11):2250‐2256. doi:10.2337/dc11-2478 22837369 PMC3476906

[22] Laxy M , Becker J , Kähm K , et al. Utility decrements associated with diabetes and related complications: estimates from a population‐based study in Germany. Value Health. 2021;24(2):274‐280. 33518034 10.1016/j.jval.2020.09.017

[23] Wan EYF , Fung CSC , Choi EPH , et al. Main predictors in health‐related quality of life in Chinese patients with type 2 diabetes mellitus. Qual Life Res. 2016;25(11):2957‐2965. 27299744 10.1007/s11136-016-1324-4

[24] Jiao F , Wong CKH , Gangwani R , Tan KCB , Tang SCW , Lam CLK . Health‐related quality of life and health preference of Chinese patients with diabetes mellitus managed in primary care and secondary care setting: decrements associated with individual complication and number of complications. Health Qual Life Outcomes. 2017;15(1):1‐12. 28610625 10.1186/s12955-017-0699-4 PMC5470199

[25] Pan C‐W , Sun H‐P , Zhou H‐J , et al. Valuing health‐related quality of life in type 2 diabetes patients in China. Med Decis Making. 2016;36(2):234‐241. doi:10.1177/0272989X15606903 26400873

[26] Mok CH , Kwok HHY , Ng CS , Leung GM , Quan J . Health state utility values for type 2 diabetes and related complications in east and Southeast Asia: a systematic review and meta‐analysis. Value Health. 2021;24(7):1059‐1067. doi:10.1016/j.jval.2020.12.019 34243830

[27] Lee WJ , Song K‐H , Noh JH , Choi YJ , Jo M‐W . Health‐related quality of life using the EuroQol 5D questionnaire in Korean patients with type 2 diabetes. J Korean Med Sci. 2012;27(3):255‐260. 22379335 10.3346/jkms.2012.27.3.255 PMC3286771

[28] Takahara M , Katakami N , Shiraiwa T , et al. Evaluation of health utility values for diabetic complications, treatment regimens, glycemic control and other subjective symptoms in diabetic patients using the EQ‐5D‐5L. Acta Diabetol. 2019;56(3):309‐319. doi:10.1007/s00592-018-1244-6 30353354

[29] Zhou Z , Jardine MJ , Li Q , et al. Effect of SGLT2 inhibitors on stroke and atrial fibrillation in diabetic kidney disease: results from the CREDENCE trial and meta‐analysis. Stroke. 2021;52(5):1545‐1556. 33874750 10.1161/STROKEAHA.120.031623 PMC8078131

[30] Gerstein HC , Hart R , Colhoun HM , et al. The effect of dulaglutide on stroke: an exploratory analysis of the REWIND trial. Lancet Diabetes Endocrinol. 2020;8(2):106‐114. doi:10.1016/S2213-8587(19)30423-1 31924562

[31] Cheung YB , Thumboo J . Developing health‐related quality‐of‐life instruments for use in Asia: the issues. Pharmacoeconomics. 2006;24(7):643‐650. doi:10.2165/00019053-200624070-00003 16802840

[32] Yao Q , Liu C , Zhang Y , Xu L . Population norms for the EQ‐5D‐3L in China derived from the 2013 National Health Services Survey. J Glob Health. 2021;11:08001. doi:10.7189/jogh.11.08001 33692898 PMC7916444

[33] Wang H , Kindig DA , Mullahy J . Variation in Chinese population health related quality of life: results from a EuroQol study in Beijing, China. Qual Life Res. 2005;14(1):119‐132. doi:10.1007/s11136-004-0612-6 15789946

[34] Morrish NJ , Wang SL , Stevens LK , Fuller JH , Keen H . Mortality and causes of death in the WHO multinational study of vascular disease in diabetes. Diabetologia. 2001;44(Suppl 2):S14‐S21. doi:10.1007/pl00002934 11587045

[35] Sullivan PW , Ghushchyan VH . EQ‐5D scores for diabetes‐related comorbidities. Value Health. 2016;19(8):1002‐1008. 27987626 10.1016/j.jval.2016.05.018

[36] Leal J , Becker F , Lim LL , Holman RR , Gray AM . Health utilities in Chinese patients with coronary heart disease and impaired glucose tolerance (ACE): a longitudinal analysis of a randomized, double‐blind, placebo‐controlled trial. J Diabetes. 2022;14(7):455‐464. 35876124 10.1111/1753-0407.13294 PMC9310045

[37] Feigin VL , Stark BA , Johnson CO , et al. Global, regional, and national burden of stroke and its risk factors, 1990–2019: a systematic analysis for the global burden of disease study 2019. Lancet Neurol. 2021;20(10):795‐820. 34487721 10.1016/S1474-4422(21)00252-0 PMC8443449

[38] Ma RC , Chan JC . Type 2 diabetes in east Asians: similarities and differences with populations in Europe and the United States. Ann N Y Acad Sci. 2013;1281(1):64‐91. doi:10.1111/nyas.12098 23551121 PMC3708105

[39] Turana Y , Tengkawan J , Chia YC , et al. Hypertension and stroke in Asia: a comprehensive review from HOPE Asia. J Clin Hypertens (Greenwich). 2021;23(3):513‐521. 33190399 10.1111/jch.14099 PMC8029540

[40] Bagust A , Beale S . Modelling EuroQol health‐related utility values for diabetic complications from CODE‐2 data. Health Econ. 2005;14(3):217‐230. doi:10.1002/hec.910 15386666

[41] Elshahat S , Cockwell P , Maxwell AP , Griffin M , O'Brien T , O'Neill C . The impact of chronic kidney disease on developed countries from a health economics perspective: a systematic scoping review. PloS One. 2020;15(3):e0230512. 32208435 10.1371/journal.pone.0230512 PMC7092970

[42] Yang F , Liao M , Wang P , Yang Z , Liu Y . The cost‐effectiveness of kidney replacement therapy modalities: a systematic review of full economic evaluations. Appl Health Econ Health Policy. 2021;19(2):163‐180. doi:10.1007/s40258-020-00614-4 33047212 PMC7902583

[43] Chan SP , Lim L‐L , Chan JC , Matthews DR . Adjusting the use of glucose‐lowering agents in the real‐world clinical Management of People with type 2 diabetes: a narrative review. Diabetes Ther. 2023;14:1‐16. 36920594 10.1007/s13300-023-01386-5 PMC10015140

[44] Nauck MA , Buse JB , Mann JF , et al. Health‐related quality of life in people with type 2 diabetes participating in the LEADER trial. Diabetes Obes Metab. 2019;21(3):525‐532. 30260088 10.1111/dom.13547 PMC6587748

[45] Look AHEAD Research Group , Wing RR , Bolin P , et al. Cardiovascular effects of intensive lifestyle intervention in type 2 diabetes. N Engl J Med. 2013;369(2):145‐154. 23796131 10.1056/NEJMoa1212914 PMC3791615

[46] Siebers M , Biedermann SV , Bindila L , Lutz B , Fuss J . Exercise‐induced euphoria and anxiolysis do not depend on endogenous opioids in humans. Psychoneuroendocrinology. 2021;126:105173. doi:10.1016/j.psyneuen.2021.105173 33582575

[47] Russo‐Neustadt A , Beard R , Huang Y , Cotman C . Physical activity and antidepressant treatment potentiate the expression of specific brain‐derived neurotrophic factor transcripts in the rat hippocampus. Neuroscience. 2000;101(2):305‐312. 11074154 10.1016/s0306-4522(00)00349-3

[48] Avena NM , Rada P , Hoebel BG . Evidence for sugar addiction: behavioral and neurochemical effects of intermittent, excessive sugar intake. Neurosci Biobehav Rev. 2008;32(1):20‐39. doi:10.1016/j.neubiorev.2007.04.019 17617461 PMC2235907

[49] Limdi JK , Hyde GM . Evaluation of abnormal liver function tests. Postgrad Med J. 2003;79(932):307‐312. doi:10.1136/pmj.79.932.307 12840117 PMC1742736

[50] Sheu WH‐H , Ji L‐N , Nitiyanant W , et al. Hypoglycemia is associated with increased worry and lower quality of life among patients with type 2 diabetes treated with oral antihyperglycemic agents in the Asia‐Pacific region. Diabetes Res Clin Pract. 2012;96(2):141‐148. 22265956 10.1016/j.diabres.2011.12.027

[51] Wieringa TH , de Wit M , Twisk JWR , Snoek FJ . Does hypoglycaemia affect the improvement in QoL after the transition to insulin in people with type 2 diabetes? J Endocrinol Invest. 2018;41(2):249‐258. doi:10.1007/s40618-017-0744-5 28803366 PMC5785617

[52] Tolerability and quality of life in ARB‐treated patients . Am J Manag Care. 2005;11(13 Suppl):S392‐S394. 16300454

[53] Mok CH , Kwok HH , Ng CS , Leung GM , Quan J . Health state utility values for type 2 diabetes and related complications in east and Southeast Asia: a systematic review and meta‐analysis. Value Health. 2021;24(7):1059‐1067. 34243830 10.1016/j.jval.2020.12.019

