# Supplementary material for: Temporal associations of diabetes‐related complications with health‐related quality of life decrements in Chinese patients with type 2 diabetes: A prospective study among 19 322 adults—Joint Asia Diabetes Evaluation (JADE) register (2007–2018)
Source: J Diabetes. 2023 Nov 20;16(6):e13503. doi: 10.1111/1753-0407.13503 (PMC11128750; doi:10.1111/1753-0407.13503)
Supplement: Supplementary file 1 — Table S1. International Classification of Diseases, Ninth Revision (ICD‐9) admissions and procedure codes for categorization of diabetes complication. Table S2. Definitions of covariates included for selection. Table S3. EuroQol 5‐dimensional questionnaire (EQ‐5D) utility in patient with diabetes. Figure S1. Annual mean estimated glomerular filtration rate (eGFR) and urinary albumin creatinine ratio (UACR) of patients with diabetes who experience different stages of chronic kidney disease. [file JDB-16-e13503-s001.docx]

**with health-related quality of life decrements in Chinese patients with type 2 diabetes: A prospective study among 19,322 adults - Joint Asia Diabetes Evaluation (JADE) Register (2007-2018)**

**Supplemental Material**

Table of contents

[Supplementary table 1. ICD-9 admissions and procedure codes for categorization of diabetes complication 2](#_Toc145933290)

[Supplementary table 2. Definitions of covariates included for selection 5](#_Toc145933291)

[Supplementary Table 3. EQ-5D utility in patient with diabetes 12](#_Toc145933292)

[Supplementary figure 1. Annual mean estimated glomerular filtration rate (eGFR) and urinary albumin creatinine ratio (UACR) of patients with diabetes who experience different stages of chronic kidney disease 21](#_Toc145933293)

# Supplementary table 1. ICD-9 admissions and procedure codes for categorization of diabetes complication

| **Coronary heart disease** | 1. Acute myocardial infarction (410) 2. Other acute and subacute forms of ischemic heart disease (411) 3. Old myocardial infarction (412) 4. Angina pectoris (413) 5. Other forms of chronic ischemic heart disease (414) |
| --- | --- |
| **Congestive heart failure** | 1) Heart failure (428) |
| **Ischemic stroke** | 1. Occlusion and stenosis of precerebral arteries (433) 2. Occlusion of cerebral arteries (434) |
| **Hemorrhagic stroke** | 1. Subarachnoid hemorrhage (430) 2. Intracerebral hemorrhage (431) 3. Other and unspecified intracranial hemorrhage (432) |
| **Amputation of lower limb** | 1. Amputation of lower limb (procedure code of 84.1) 2. Discharges with a traumatic amputation such as automobile accidents (895-897) were excluded |
| **Peripheral vascular disease (excluding amputation of lower limb)** | 1. Diabetes with peripheral circulatory disorders (250.7) 2. Gangrene (785.4) 3. Peripheral angiopathy in diseases classified elsewhere (443.81) 4. Peripheral vascular disease, unspecified (443.9) 5. Procedures: Other (peripheral) vascular shunt or bypass (procedure code 39.29); insertion of non-drug-eluting peripheral vessel stent(s) (39.90); 38.08, 38.18, 38.38, 38.48, 38.68, 38.88, 39.25, 39.49, 39.56, 39.57, 39.58, 39.59, 39.99; 00.55, 17.56, 39.50, 39.79 |
| **Chronic kidney disease (CKD)** | Defined according to the Kidney Disease: Improving Global Outcomes (KDIGO) guidelines, CKD was classified based on estimated glomerular filtration rate (eGFR) category into 5 stages (G1-G5). CKD G3 and 4 (GFR 59-15 ml/min/1.73m^2^) represent loss of 50% or more of normal kidney function and are seen as a cut-off for clinically significant CKD, whereas G5 covers GFR under 15 ml/min/1.73m^2 51^. |
| **CKD G3-4 without dialysis nor transplant** | 2 measures of egfr<15 ml/min/1.73m^2^separated by 90-365 days  Excluding the following ICD-9 / procedure codes:   1. Hemodialysis dialysis (procedure code 39.95) with diagnosis of Chronic kidney disease (585) or Renal failure (586) 2. Peritoneal dialysis (procedure code 54.98) 3. Transplant of kidney (procedure code 55.6) 4. Complications of transplanted kidney (996.81) 5. Persons with a condition influencing their health status; organ or tissue replaced by transplant; kidney (V42.0) |
| **End stage kidney disease CKD G5** | 2 measures of egfr<15 ml/min/1.73m^2^separated by 90-365 days  **With dialysis:**   1. Hemodialysis dialysis (procedure code 39.95) with diagnosis of Chronic kidney disease (585) or Renal failure (586) 2. Peritoneal dialysis (procedure code 54.98)   **With kidney transplant:**   1. Transplant of kidney (procedure code 55.6) 2. Complications of transplanted kidney (996.81) 3. Persons with a condition influencing their health status; organ or tissue replaced by transplant; kidney (V42.0) |
| **Cancer** | Neoplasms (140-208) |

# Supplementary table 2. Definitions of covariates included for selection

| **Covariates** | **Categories (if categorical)** | **Remarks** |
| --- | --- | --- |
| **Demographics and lifestyle** |  |  |
| Sex | Male  Female |  |
| Age (years old) | < 40  40-59  ≥ 60 |  |
| Age at diagnosis (years old) | < 40  40-59  ≥ 60 |  |
| Number of years with diabetes (years) | <5  5 - 9  10 - 14  ≥ 15 |  |
| Type of care | Public  Private |  |
| Year of doctor’s visit (survey completion year) | Between 2007-2018 |  |
| Education level | Primary, illiterate or Others  Middle or high school  College or Above |  |
| Use of alcohol | Never  Occasional  Regular  Ex-drinker |  |
| Use of smoking | Never  Yes  Ex-smoker |  |
| frequency of physical activity (times/week) | No regular activity  <3  3-4  5  >5 | Vigorous exercise corresponding to brisk walking of > 30 minutes |
| Adherence to balanced diet in last 3 months | Never  No  Occasional  Yes |  |
| **Clinical and biochemical characteristics** |  |  |
| Body mass index (BMI) (kg/m2) | <25  25 - 30  ≥ 30 or more |  |
| Waist hip ratio | < 0.85  ≥ 0.85 < 0.90  ≥ 0.90 |  |
| Diastolic blood pressure (mmHg) | < 75  ≥ 75 < 85  ≥ 85 |  |
| Systolic blood pressure (mmHg) | < 125  ≥ 125 < 140  ≥ 140 |  |
| High-density lipoprotein (HDL) cholesterol (mmol/L) | < 1.3  ≥ 1.3 < 1.55  ≥ 1.55 |  |
| Low-density lipoprotein (LDL) cholesterol (mmol/L) | <2.6  ≥ 2.6 < 3.35  ≥ 3.35 |  |
| Triglycerides (mmol/L) | < 1.69  ≥ 1.69 < 2.26  ≥ 2.26 |  |
| HbA1c (%) | < 5.7  ≥ 5.7 < 7.0  ≥ 7.0 |  |
| Fasting plasma glucose (FPG) (mmol/L) | <5.7  ≥ 5.7 < 7.0  ≥ 7.0 |  |
| Estimated glomerular filtration rate (ml/min/1.73m2) | < 30  ≥ 30 < 60  ≥ 60 < 90  ≥ 90 | Calculated using the CKD-EPI formula |
| Urinary albumin creatinine ratio (UACR) | < 30  ≥30 <300  ≥ 300 |  |
| Plasma albumin (g/L) | < 35  ≥35 <50  ≥ 50 |  |
| Alanine aminotransferase (mmol/L) | < 10  ≥ 10 < 40  ≥ 40 |  |
| Alkaline phosphatase (mmol/L) | < 44  ≥ 44 < 147  ≥ 147 |  |
| Bilirubin (µmol/L) | < 5  ≥ 5 < 21  ≥ 21 |  |
| Haematocrit | < 0.40  ≥ 0.40 < 0.50  ≥ 0.50 |  |
| Haemoglobin (g/dL) | < 10  ≥ 10 |  |
| **Medical treatments** |  |  |
| Glucose lowering drugs |  | Alpha-glucosidase inhibitor (AGIs), dipeptidyl peptidase 4 (DPP-4) inhibitor, meglitinide, metformin, sodium-glucose cotransporter-2 (SGLT2) inhibitor, sulphonylurea, thiazolidinedione |
| Angiotensin-converting enzyme (ACE) inhibitor |  |  |
| angiotensin receptor blocker (ARB) inhibitor |  |  |
| Blood pressure lowering drugs (excluding RAS inhibitors – ACE/ARB) |  | Alpha blocker, beta blocker, calcium channel blocker, hydralazine, methyldopa, nitrate, thiazide |
| Statins |  |  |
| Insulin |  |  |
| **Medical history** |  |  |
| frequency of self-reported hypoglycaemia in the past 3 months | None Less than once monthly  At least once monthly  At least once weekly  At least daily | Sweating, tremor, feeling of hunger especially if reversed by food |
| Sensory neuropathy | None  Yes | Two of three abnormal features: symptoms (subjective) in the feet, and signs (objective) by reduced vibration sensation to tuning fork and reduced pressure sensation to monofilament |
| Visual acuity (VA) | Severe visual impairment (counting fingers, hand movement, light perception)  Blind (no light perception) | Measured by Snellen chart corrected by glasses or pinhole. Severe visual impairment short of blindness with no light perception was defined as VA below 20/200 categorised by counting fingers, observing hand movement or light perception |
| Diabetic retinopathy | Non-proliferative/ pre-proliferative retinopathy  Severe retinopathy | Retinal photography was used to define diabetic retinopathy read by endocrinologists or trained fellows. Severe retinopathy was defined as proliferative retinopathy, maculopathy or advanced eye disease. |
| Eye treatment | None  Yes | Cataract, retinal and laser surgery |
| Elixhauser co-morbidity score | <0  0  >0 <5  ≥5 <15  ≥15 <25  ≥25 |  |

# Supplementary Table 3. EQ-5D utility in patient with diabetes

| **Variable** | **Estimates** | **P-value** |
| --- | --- | --- |
| **(Intercept)** | 0.881 ( 0.865, 0.897) | <0.001** |
| **Congestive heart failure (ref: none)** |  |  |
| < 1 year | -0.061 (-0.085,-0.037) | <0.001** |
| 1-2 years ago | -0.075 (-0.105,-0.045) | <0.001** |
| 2-3 years ago | -0.065 (-0.101,-0.029) | <0.001** |
| >3 years ago | 0.023 (-0.002, 0.049) | 0.074 |
| **Ischaemic stroke (ref: none)** |  |  |
| < 1 year | -0.165 (-0.191,-0.139) | <0.001** |
| 1-2 years ago | -0.126 (-0.159,-0.093) | <0.001** |
| >2 years ago | -0.081 (-0.095,-0.067) | <0.001** |
| **Haemorrhagic stroke (ref: none)** |  |  |
| < 1 year | -0.230 (-0.289,-0.170) | <0.001** |
| >1 year ago | -0.125 (-0.155,-0.096) | <0.001** |
| **Lower extremity amputation (ref: none)** |  |  |
| Any year | -0.093 (-0.126,-0.059) | <0.001** |
| **Peripheral artery disease (ref: none)** |  |  |
| < 1 year | -0.117 (-0.165,-0.070) | <0.001** |
| >1 year ago | 0.005 (-0.025, 0.035) | 0.752 |
| **Chronic kidney disease** |  |  |
| **Stage 3-4 without dialysis nor kidney transplant (ref: none)** |  |  |
| Same year | -0.042 (-0.052,-0.032) | <0.001** |
| >1 year ago | -0.022 (-0.031,-0.012) | <0.001** |
| **End stage kidney disease** |  |  |
| **G5 without dialysis without kidney transplant (ref: none)** |  |  |
| Any year | -0.079 (-0.135,-0.023) | 0.006** |
| **Sensory neuropathy (ref: none)** | -0.089 (-0.098,-0.080) | <0.001** |
| **Diabetic retinopathy (ref: none)** |  |  |
| Non-proliferative/ pre-proliferative retinopathy | 0.006 ( 0.001, 0.012) | 0.032* |
| Severe retinopathy | 0.003 (-0.007, 0.012) | 0.547 |
| **Visual acuity (ref: no visual impairment)** |  |  |
| Severe visual impairment (counting fingers, hand movement, light perception) | -0.016 (-0.027,-0.005) | 0.004** |
| Blind (no light perception) | -0.052 (-0.079,-0.025) | <0.001** |
| **History of eye treatment (ref: none)** |  |  |
| Cataract, retinal and laser surgery | -0.007 (-0.014,-0.001) | 0.025* |
| **Elixhauser co-morbidity score (Ref: score 0)** |  |  |
| <0 | 0.005 (-0.008, 0.018) | 0.422 |
| >0 <5 | -0.009 (-0.018,-0.001) | 0.032* |
| ≥5 <15 | -0.007 (-0.013, 0.000) | 0.035* |
| ≥15 <25 | -0.020 (-0.033,-0.008) | <0.001** |
| ≥25 | -0.049 (-0.087,-0.011) | 0.012* |
| **Personal characteristics** |  |  |
| **Sex (ref: male)** | -0.043 (-0.048,-0.037) | <0.001** |
| **Age (years old) (ref: 40-59)** |  |  |
| < 40 years old | 0.009 (-0.004, 0.022) | 0.193 |
| ≥ 60 years old | -0.002 (-0.008, 0.004) | 0.519 |
| **Age at diagnosis (years old) (ref: 40-59)** |  |  |
| < 40 | 0.014 ( 0.006, 0.022) | <0.001** |
| ≥ 60 | -0.022 (-0.029,-0.015) | <0.001** |
| **Number of years with diabetes (years) (ref: <5 years)** |  |  |
| 5 - 9 years | -0.008 (-0.014,-0.002) | 0.011* |
| 10 - 14 years | -0.018 (-0.025,-0.010) | <0.001** |
| ≥ 15 years | -0.030 (-0.038,-0.021) | <0.001** |
| **Year of doctor's visit (survey completion year)** | 0.001 ( 0.000, 0.002) | 0.049* |
| **Type of care (ref: Public)** |  |  |
| Private | 0.005 (-0.002, 0.013) | 0.141 |
| **Education (ref: middle or high school)** |  |  |
| Primary, illiterate or Others | -0.006 (-0.011,-0.001) | 0.029* |
| College or Above | 0.001 (-0.006, 0.008) | 0.741 |
| **Physical activity level (ref: no regular activity)** |  |  |
| <3 times /week | 0.019 ( 0.012, 0.026) | <0.001** |
| 3-4 times /week | 0.022 ( 0.014, 0.030) | <0.001** |
| 5 times / week | 0.035 ( 0.024, 0.046) | <0.001** |
| >5 times / week | 0.029 ( 0.023, 0.034) | <0.001** |
| **Frequency of hypoglycemia (ref: none)** |  |  |
| Less than once monthly | -0.011 (-0.018,-0.004) | 0.002** |
| At least once monthly | -0.029 (-0.038,-0.020) | <0.001** |
| At least once weekly | -0.044 (-0.059,-0.030) | <0.001** |
| At least daily | 0.007 (-0.045, 0.060) | 0.781 |
| **Use of alcohol (ref: never)** |  |  |
| Occasional | 0.009 ( 0.004, 0.015) | 0.001** |
| Regular | 0.008 (-0.005, 0.020) | 0.241 |
| Ex-drinker | -0.011 (-0.018,-0.003) | 0.004** |
| **Smoking (ref: never)** |  |  |
| Current | -0.004 (-0.012, 0.003) | 0.252 |
| Ex-smoker | -0.005 (-0.011, 0.002) | 0.16 |
| **Body mass index (kg/m^2^) (Ref: <25)** |  |  |
| 25 - 30 | -0.001 (-0.006, 0.004) | 0.61 |
| ≥ 30 or more | -0.023 (-0.030,-0.017) | <0.001** |
| **HDL-cholesterol (mmol/L) (Ref: ≥ 1.55)** |  |  |
| < 1.3 | -0.012 (-0.018,-0.007) | <0.001** |
| ≥ 1.3 < 1.55 | -0.004 (-0.010, 0.002) | 0.236 |
| **LDL-cholesterol (mmol/L) (Ref: <2.6)** |  |  |
| ≥ 2.6 < 3.35 | 0.009 ( 0.004, 0.014) | 0.001** |
| ≥ 3.35 | 0.007 ( 0.000, 0.014) | 0.05 |
| **Diastolic blood pressure (mmHg) (ref:< 75)** |  |  |
| ≥ 75 < 85 | -0.006 (-0.010,-0.001) | 0.030* |
| ≥ 85 | -0.009 (-0.015,-0.003) | 0.002** |
| **Hemoglobin A1C (%) (ref:< 5.7)** |  |  |
| ≥ 5.7 < 7.0 | 0.018 ( 0.005, 0.031) | 0.005** |
| ≥ 7.0 | 0.023 ( 0.010, 0.036) | <0.001** |
| **Fasting plasma glucose (mmol/L) (ref: <5.7)** |  |  |
| ≥ 5.7 < 7.0 | 0.007 ( 0.000, 0.014) | 0.036* |
| ≥ 7.0 | 0.013 ( 0.006, 0.019) | <0.001** |
| **Estimated glomerular filtration rate (ml/min/1.73m^2^) (Ref: ≥ 90)** |  |  |
| < 30 | 0.007 (-0.006, 0.020) | 0.289 |
| ≥ 30 < 60 | 0.016 ( 0.007, 0.025) | <0.001** |
| ≥ 60 < 90 | 0.001 (-0.004, 0.007) | 0.692 |
| **Plasma albumin (g/L) (Ref: ≥35 <50)** |  |  |
| < 35 | -0.074 (-0.092,-0.057) | <0.001** |
| ≥ 50 | 0.002 (-0.015, 0.018) | 0.822 |
| **Alanine transaminase levels (IU/L) (Ref: ≥ 10 < 40)** |  |  |
| < 10 | -0.025 (-0.038,-0.011) | <0.001** |
| ≥ 40 | 0.000 (-0.006, 0.006) | 0.93 |
| **Haemoglobin (g/dl) (Ref: ≥ 10)** |  |  |
| < 10 | -0.022 (-0.036,-0.008) | 0.003** |
| **Existing treatments (Ref: none)** |  |  |
| Glucose lowering drugs | 0.009 ( 0.002, 0.016) | 0.011* |
| Insulin | -0.001 (-0.007, 0.005) | 0.808 |
| Angiotensin converting enzyme (ACE) inhibitors | 0.011 ( 0.005, 0.017) | <0.001** |
| Angiotensin receptor blockers (ARB) | -0.006 (-0.014, 0.001) | 0.103 |
| Anti-hypertensives (excluding ACE inhibitors/ARB) | -0.002 (-0.008, 0.005) | 0.594 |
| Statins | -0.005 (-0.010, 0.000) | 0.039* |

*Note: The mean EQ-5D utility was 0.880 for a ‘referent patient’ with the following profile: a Chinese man of 40-59 years and age of diagnosis of 40-59 years who underwent assessment in 2012 in a public hospital clinic setting, with disease duration less than 5 years and education level of middle or high school. He did not drink alcohol, smoke or perform regular exercise and had not experienced hypoglycaemia in the 3 months prior to assessment. He did not have any complication with a co-morbidity score of 0. His BMI was less than 25 kg/m^2^ and diastolic BP less than 75 mmHg. His HDL-cholesterol was ≥1.55 mmol/L, LDL-cholesterol, <2.6 mmol/L, HbA_1C_<5.7%, FPG <5.7mmol/L and eGFR ≥90 mL/min/1.73m^2^. His plasma albumin range was 35-50 g/L, alanine transaminase, 10-40 IU/L, blood haemoglobin≥ 10g/dL and he was not on any drug treatment*

# Supplementary figure 1. Annual mean estimated glomerular filtration rate (eGFR) and urinary albumin creatinine ratio (UACR) of patients with diabetes who experienced different stages of chronic kidney disease


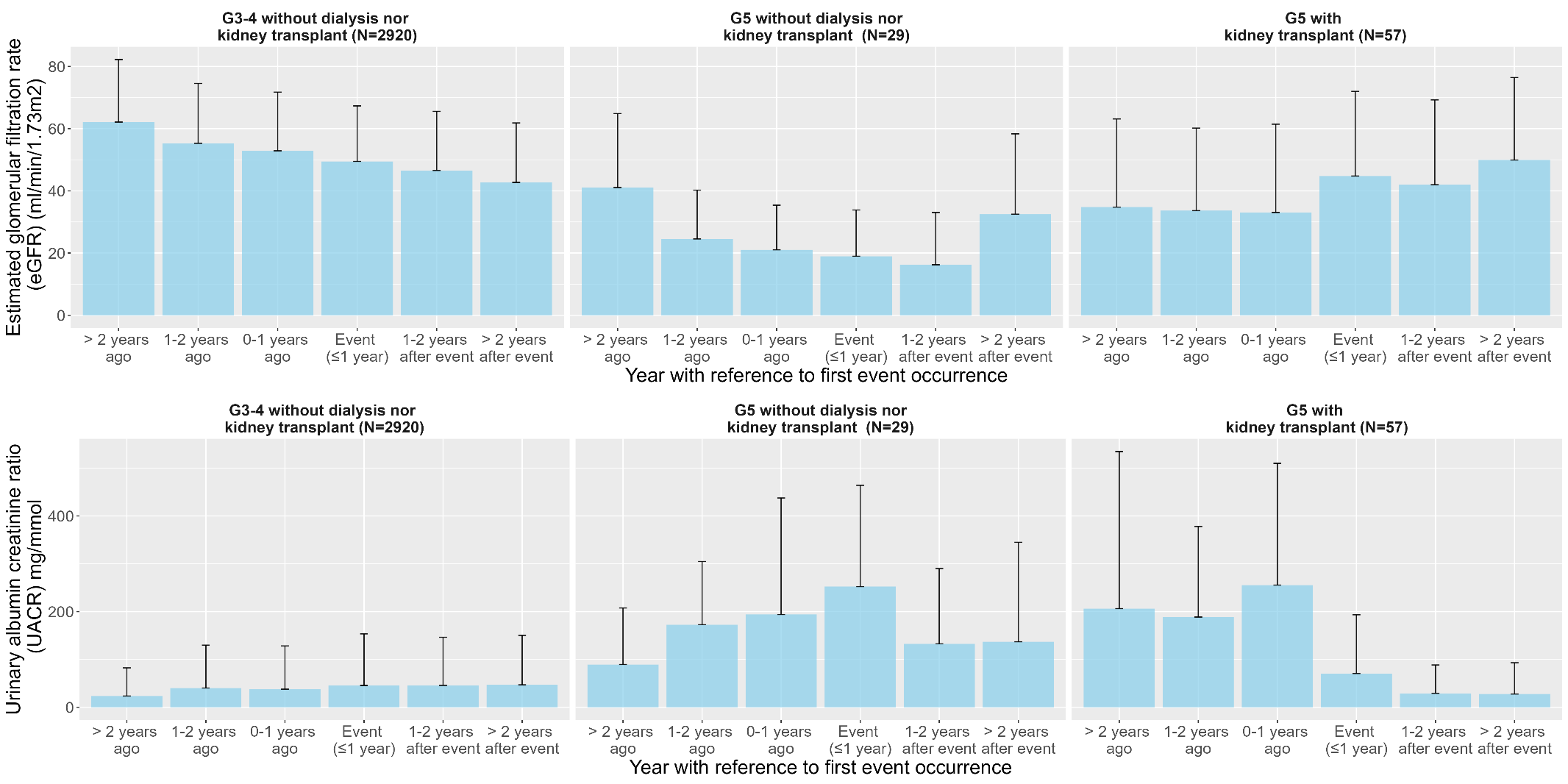


*Note: eGFR measures how effectively the kidneys are filtering waste products from the blood. According to the Kidney Disease: Improving Global Outcomes (KDIGO) guidelines*^51^*, eGFR is categorized into stages ranging from G1 to G5, representing different levels of kidney function decline. The eGFR is categorized as follows- G1: eGFR >= 90 mL/min/1.73m² (normal or high kidney function), G2: eGFR 60-89 mL/min/1.73m², G3a: eGFR 45-59 mL/min/1.73m², G3b: eGFR 30-44 mL/min/1.73m², G4: eGFR 15-29 mL/min/1.73m², G5: eGFR < 15 mL/min/1.73m² (kidney failure requiring dialysis or transplantation). UACR, measures the amount of albumin (a protein) present in the urine relative to creatinine levels. It is used to assess kidney damage and the presence of proteinuria. The UACR is categorized as follows- A1 (normal to mildly increased): UACR < 3 mg/mmol, A2 (moderately increased): UACR 3-30 mg/mmol, and A3 (severely increased) UACR: >30 mg/mmol.*
